# Facial expressions during compound interventions of nociception, conspecific isolation, and sedation in horses

**DOI:** 10.1038/s41598-025-89329-x

**Published:** 2025-02-13

**Authors:** Johan Lundblad, Marie Rhodin, Elin Hernlund, Hanna Bjarnestig, Sara Hidén Rudander, Pia Haubro Andersen

**Affiliations:** Department of Animal Biosciences, Swedish University of Agricultural Sciences, PO Box 7023, Uppsala, 750 07 Sweden

**Keywords:** Animal behaviour, Predictive medicine, Animal physiology

## Abstract

Facial expressions in prey animals such as equines can convey information regarding their internal state and are therefore often used as cues for welfare and pain evaluation. The state of pain is commonly compared to a neutral state with little consideration given to other experiences that could affect the animal, although this situation is rare outside of experimental contexts. To evaluate the effect of managerial contexts on facial expressions from a nociceptive input, conspecific isolation and sedation with known physiological effects were compared to compound states of nociception. Using an anatomically based facial action coding system and a short acting pain model, patterns of facial activity could discriminate between horses experiencing conspecific isolation, sedation, and a nociceptive stimulus separately. Nociception occurring together with conspecific isolation could not be discriminated from the conspecific isolation alone, and compound nociception and sedation could not be discriminated from control. While blinking frequency demonstrated potential to be a valuable marker when evaluating a nociceptive stimulus in sedated horses, careful consideration must be given to the biological interpretation of facial expressions during situations where managerial or drug effects may be present.

**Supplementary Information:**

The online version contains supplementary material available at 10.1038/s41598-025-89329-x.

## Introduction

Developing methods to understand an animal’s subjective experiences within different contexts is a challenging but important endeavor. Facial expressions in mammals are widely recognized as significant conveyors of information to conspecifics about subjective states and motivation^1^. Indeed, the incorporation of facial expressions into animal welfare assessments has been proposed^2^, as they offer valuable insights into an animal’s perception of its situation, a unique aspect not captured by other welfare measures. Among the communication strategies in humans, facial expressions are one of the most prominent communication tools^3^. Research on animal facial repertoires is less extensive, but some studies have examined pain^4–7^ and various other contexts, such as contentment^8^, frustration^9^, anticipation^10^, and physiological stress^11^ in animals. Horses possess a particularly expressive facial repertoire^12^, surpassing that of dogs^13^ or chimpanzees^14^, making them promising models for an investigation into animal facial expressions. In equines, studies have observed increased occurrence rates of expressions such as backward-rotated ears, dilated nostrils, and muscle contractions in the mental region and above the eye, resulting in tense muscles around the lower face and an eye wrinkle during experimentally induced pain^15^. Similar facial markers of pain have been identified independently to develop grimace-based pain scales for horses under clinical conditions^16,17^.

Interestingly, recent findings indicate that routine, non-painful management procedures may induce changes in the equine facial repertoire akin to expressions observed during pain, including dilated nostrils, increased blinking, and wrinkles above the upper eyelid^11^. In contrast, content and relaxed horses tend to exhibit asymmetrical ears, protruding lips, and half-closed eyes^8^. Horses injected with sedative drugs, which are commonly used during management and clinical procedures to mitigate aversive behaviors, also display similar facial activity^18^. These discoveries thus challenge the notion that specific facial displays are distinctly correlated with specific situations. Instead, the dynamic nature of facial expressions in relation to shifting or continual contexts remains unclear. This issue is yet to be confronted prior to the inclusion of facial expressions in pain and welfare assessments. A direct assessment of individual facial expressions in horses is complicated by their tendency to suppress or diminish certain behaviors when observed by humans^19^, underscoring the importance of context and circumstances within the observation.

Existing knowledge about facial expressions and their cause largely stems from human research, where self-reporting is frequently used as a proxy for the underlying motivation behind the facial movement^20^. The understanding of facial expressions in humans may be partly transferable to animals, considering the similarity of activation of facial muscles during pain that has been measured across species^21^. This would indicate evolutionary preservation of at least some of the neuroanatomical processes responsible for facial muscle contractions. However, there are certain notable differences between human and animal studies when studying these pathways. While the study of facial language in humans has a significant upper hand due to the ability of verbal self-report, it still presents challenges and often requires complementary markers^22^. Animal studies must rely fully on such markers, which often place emphasis on physiological markers and behavioral observations. A drawback of behavioral studies is bias from researchers’ subjective perceptions and expectancy biases rooted in other cues or knowledge regarding the animal’s state^23^. Observation of contractions of single well-defined muscles is thought to be less prone to bias, and also facilitates consistent recordings of animal facial expressions. The Facial Action Coding System (FACS)^24^ offers a standardized and comparable method. It categorizes facial expressions into Action Units (AUs), representing morphological facial changes caused by muscle contractions governed by the facial nerve. Action Descriptors (ADs) represent less precise or simultaneous multiple muscle contractions that affect facial morphology. While initially designed for human facial activity, this system has been adapted for eight species^12–14,25–29^.

While FACS has been successfully used to describe the facial displays of pain^30,31^ and stress^11^ in horses, little is known about the facial expressions when two different stimuli, such as nociception and stress, occur together in compound situations. The purpose of this study was therefore to evaluate if facial expressions can still be used to distinguish discrete states when they co-occur. The hypothesis was that equine facial expressions are affected by these discrete interventions, and can be distinguished by data driven methods, but not when the same interventions occur together.

An experimental within-subject, semi-randomized cross-over study (*n* = 12) was developed to investigate the effect of short, controlled interventions which are likely to stimulate both emotional and physiological components on facial muscle contractions in the horse. The interventions involved a non-invasive, reversible nociceptive stimuli (a stimulus transduced and encoded by nociceptors^32^), social isolation, and pharmacological sedation, contexts which have an effect on both physiology^33–35^ and emotional experience^32,36^ but stem from completely different inputs. Facial activity was coded according to the equine specific FACS^12^ and FACS data during controlled interventions and compound states (nociceptive input combined with sedation or with isolation from conspecifics) were compared within a 30-s observation window in each intervention. These interventions and their specific hypothesis are detailed in Table 1. While the focus of the study was on the contextual intervention, emotional experiences are discussed around the arousal and valence model^37^. Valence, which indicates the level of pleasantness of the intervention, was not specifically measured in this experiment but it is generally considered negative for isolation (as horses prefer the company of conspecifics^38^) and nociception^32^. Arousal, the level of physiological activity, was measured using conventional physiological markers, primarily to support the experimental design regarding the isolation intervention. A partial least squares discriminant analysis (PLS-DA^39^) was used to assess whether facial expressions, either collectively or alone, can be used to differentiate between different discrete and compound contexts.

**Table 1:** Overview of study interventions and scientific expectations.

Intervention	Induction method	Expected level of arousal	Hypothesis for facial activity
Control (C)	Calm environment with conspecific present	Low	–
Nociception (N)	Ischemia of the forearm	Unchanged	Facial activity similar to earlier reported studies of pain
Isolation (I)	Isolation from conspecifics	Increased	Increased level of facial activity similar to earlier reported studies
Sedation (S)	Intravenous injection of a non-analgesic sedative	Diminished	Diminished level of facial activity similar to earlier reported studies
Compound Sedation and Nociception (SN)	Injection of sedative combined with ischemia of the forearm	Diminished	Sedation diminishes facial activity present during Nociception making discrimination between Nociception and SN difficult
Compound Nociception and Isolation (NI)	Ischemia of the forearm combined with isolation form conspecifics	Increased	Isolation overshadows facial activity present during Nociception making discrimination between Nociception and NI difficult

## Results

### Classification of interventions

**Fig. 1:**
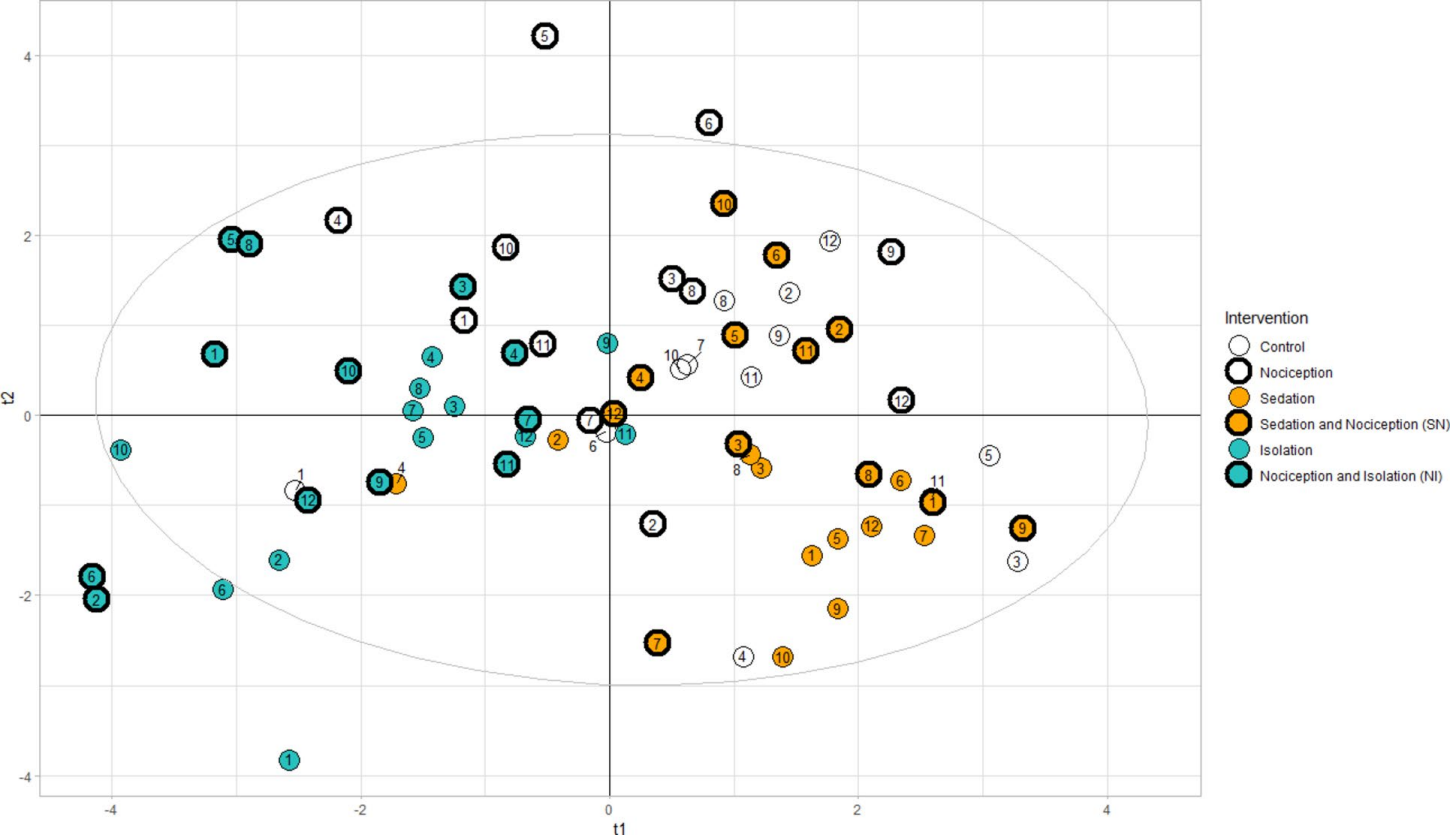
Score plot of the two first components in the PLS-DA. Each point corresponds to an individual horse (*n* = 12), colored according to intervention and plotted as a scatterplot over orthogonal new variables (components t1 and t2) consisting of weighted summaries of the frequencies of EquiFACS-codes designed to explain as much variation as possible while simultaneously separating the interventions as much as possible. The ellipse indicates a 95% confidence interval.

A total of 1287 AU events (onset to offset) and 1267 AD events were coded according to EquiFACS for 30-s episodes over the five interventions for the 12 participating horses. Using all EquiFACS-codes as variables in the PLS-DA, two components (t1, t2) consisting of weighted variables were modeled (Fig. 1). The number of included components was selected subjectively based on ease of interpretation and the cumulative Q^2^-value. This was negative for more than two components, meaning that the inclusion of additional components would have caused the model to perform less effectively. A total of 19% of variation in the dataset was explained by the two first components. The contribution of component t1 to the model was significant (Q^2^ > 0.05), while t2 was not. Interventions involving isolation were successfully differentiated from interventions involving sedation and control along the t1-axis. The scores for the nociception intervention showed the greatest amount of variation according to the t1-component but were concentrated in the top part of the scores plot along with the t2-component (Fig. 1). The only intervention that could not be clearly differentiated from the control intervention was sedation combined with simultaneous nociception, which clustered in the top right corner of the scores plot together with the control. Isolation with and without nociception present clustered proximate to each other with only a slight difference, displaying low discriminatory power.

**Fig. 2:**
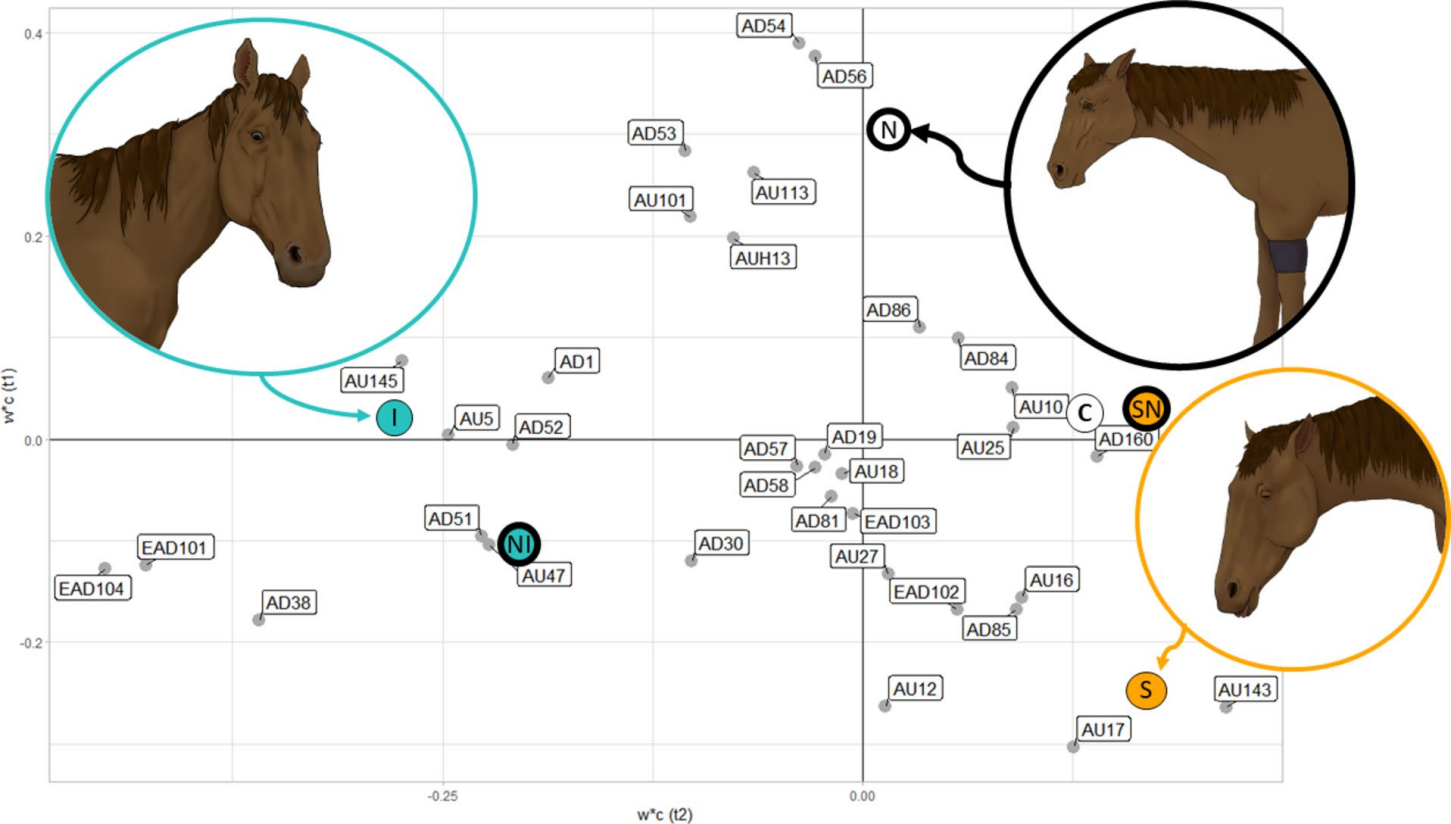
Weight scatter plot (w*c(t1), w*c(t2)). Presenting the relation between EquiFACS codes (grey) and indicator variables of the interventions (isolation (I), sedation (S), nociception (N), compound nociception and isolation (NI), compound nociception and sedation (SN), and control (C)). EquiFACS codes scattered in the proximity of the interventions have the highest discriminatory power between interventions. Variables in proximity of each other are considered correlated. Illustration by I. Lundblad.

**Fig. 3:**
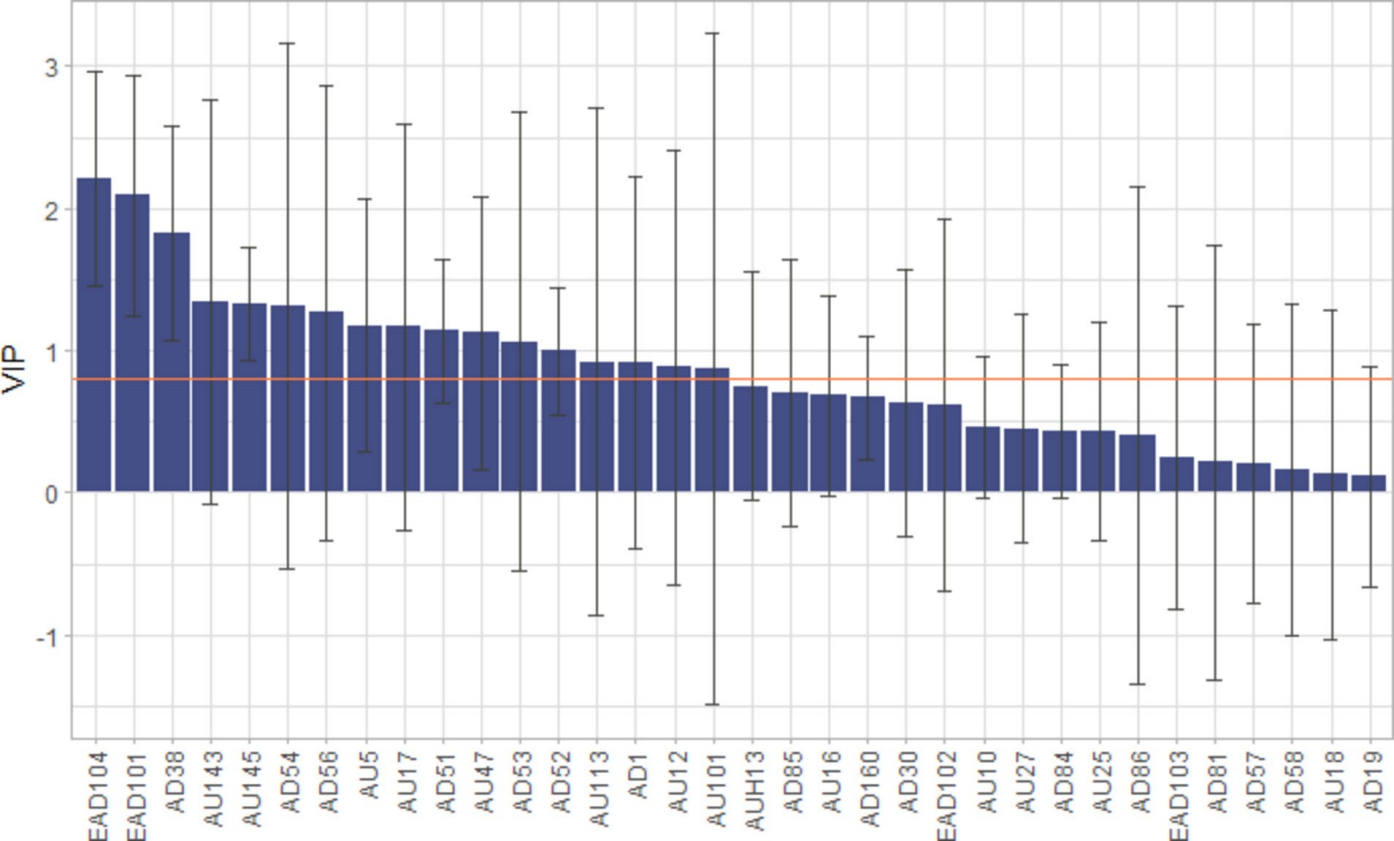
Variable importance in projection (VIP) plot. VIP-values were calculated by adding the sums of squares of the PLS loading weights adjusted with the number of explained sums of squares of each component (t1, t2), representing the contribution of each EquiFACS code to the two PLS-DA components. The average VIP is standardized to 1. Codes with values > 0.8 (orange line) were retained when testing for inference. Error bars indicate a 95% confidence interval of VIP values.

The loadings plot from the PLS-DA (Fig. 2) revealed the AUs that contributed the most to each intervention (indicator variable) that the horse experienced. In Fig. 2, variables (grey) corresponding to facial expressions according to EquiFACS that are closer to each other or closer to the indicator variables indicate positive correlations, while data points on the inverse quadrant indicate negative correlation. The AUs clustered around the isolation indicator variables were: ear movements (EAD101 & EAD104), nostril dilator (AD38), blink and half blink (AU145 & AU47), upper lid raiser (AU5), and lateral head movements (AD51 & 52). The AUs clustered around the sedation and control indicator variables predominantly related to the lower face, i.e., chin raiser (AU17), lower lip depressor (AU16), lip corner puller (AU12), and lower lip relax (AD160). For the sedation intervention, eye closure (AU143) was also prominent. The AUs clustered around the nociception indicator variable were: inner brow raiser (AU101), nostril lift (AUH13), and vertical head movements (AD53-54). The estimated importance of each AU in the discriminatory model is displayed in Fig. 3 where only AUs with variable importance in a projection above 0.8 were retained for statistical testing.

### Changes in facial activity

From the set of facial expressions represented by codes with a VIP score above 0.8, six individual codes exhibited significant variation between interventions when all interventions were included. These were: ear movements (EAD101, EAD104, *p* < 0.001), nostril dilator (AD38, *p* < 0.001), blinking (AU145, *p* < 0.001), chin raiser (AU17, *p* = 0.04), and inner brow raiser (AU101, *p* < 0.01) and were therefore included in contrast testing. Model estimates for the ratio of the number of specific EquiFACS codes between baseline and intervention are presented in Table 2. In general, facial activity showed the greatest differences compared with the control. Blinking (AU145) increased significantly during the interventions containing social isolation and the interventions containing nociception (N, SN) but remained unchanged during sedation. The only significant change between the nociception intervention and control was an increase in blinking (AU145), which occurred in all interventions that involved nociception or isolation. Two ADs, nostril dilator (AD38) and ear movements (EAD101 and EAD104), increased during the isolation interventions, but not in the any of the other interventions. For inner brow raiser (AU101), chin raiser (AU17), upper lid raiser (AU5), and eye closure (AU143), no significant difference from the control was observed.

**Table 2:** Estimated ratios for specific EquiFACS codes.

AU/AD	Contrast	Ratio (compared with Control)	SE	*p*
EAD104	Nociception	1.33	0.33	0.632
EAD104	Sedation	1.19	0.31	0.942
EAD104	Sedation and Nociception	0.55	0.17	0.223
**EAD104**	**Isolation**	**2.38**	**0.49**	**< 0.001**
**EAD104**	**Nociception and Isolation**	**2.41**	**0.49**	**< 0.001**
EAD101	Nociception	1.41	0.35	0.541
EAD101	Sedation	1.11	0.28	0.992
EAD101	Sedation and Nociception	0.67	0.19	0.529
**EAD101**	**Isolation**	**2.49**	**0.55**	**< 0.001**
**EAD101**	**Nociception and Isolation**	**2.65**	**0.58**	**< 0.001**
AD38	Nociception	3.61	3.04	0.249
AD38	Sedation	4.25	3.37	0.154
AD38	Sedation and Nociception	5.07	4.04	0.097
**AD38**	**Isolation**	**18.58**	**14.39**	**< 0.001**
**AD38**	**Nociception and Isolation**	**20.84**	**15.30**	**< 0.001**
**AU145**	**Nociception**	**1.61**	**0.30**	**0.046**
AU145	Sedation	1.06	0.23	0.999
**AU145**	**Sedation and Nociception**	**1.77**	**0.33**	**0.008**
**AU145**	**Isolation**	**1.93**	**0.35**	**0.001**
**AU145**	**Nociception and Isolation**	**2.37**	**0.41**	**< 0.001**
AU17	Nociception	1.21	0.65	0.995
AU17	Sedation	2.34	0.99	0.155
AU17	Sedation and Nociception	2.39	1.02	0.145
AU17	Isolation	2.16	0.98	0.281
AU17	Nociception and Isolation	0.70	0.39	0.941
AU101	Nociception	2.41	0.87	0.053
AU101	Sedation	1.45	0.62	0.800
AU101	Sedation and Nociception	0.72	0.40	0.947
AU101	Isolation	1.53	0.59	0.644
AU101	Nociception and Isolation	1.14	0.45	0.995

Statistical tests were performed on the logarithmic scale and values shown are back-transformed. p-values were corrected according to the multivariate t method.

SE = standard error.

Ratios are calculated between the control and the different interventions according to the ZI-GLMM model, where a value of 1 indicates no difference and bold type indicates a significant difference.

### Heart rate

Mean heart rates of each individual horse during the different interventions are presented in Table 3. There was no difference between control and nociception (t = 0.751). Mean heart rate increased during sedation, isolation, and compound interventions (C-S: t = 2.462, C-I: t = 2.646, C-SN: t = 2.967, C-NI: t = 2.676). Heart rate variability, indicated by the Root Mean Square of Successive Differences (RMSSD), was significantly larger in the Isolation intervention compared to the control (t = 2.499, *p* = 0.0295) but remained unchanged for all the other interventions. The orders of the isolation interventions had no significant effect on the results.

**Table 3:** Heart rate and heart rate variability parameters of the participating horses during the different interventions.

	Control (*n* = 12)	Nociception (*n* = 12)	Sedation (*n* = 12)	SN (*n* = 11)	Isolation (*n* = 12)	NI (*n* = 11)
Mean HR (bpm)	38.0	39.7	44.4*	43.4*	52.2*	47.8*
HR range (bpm)	29.7–44.2	30.4–64.0	36.0–61.5	36.9–55.0	31.5–92.2	31.1–72.7
Mean RMSSD (ms)	82.7	124.8	87.0	130.8	182.5*	99.4
RMSSD range (ms)	46.8–159.3	52.4–389.6	24.4–187.0	44.3–409.8	34.8–527.5	29.0–225.7

Data is presented as a mean and range over the full intervention.

*HR* heart rate presented in beats per minute (bpm), *RMSSD* root mean square of successive differences (marker of heart rate variability) presented in milliseconds (ms).

Asterisks indicate significance (*p* < 0.05).

### Serum cortisol

**Fig. 4:**
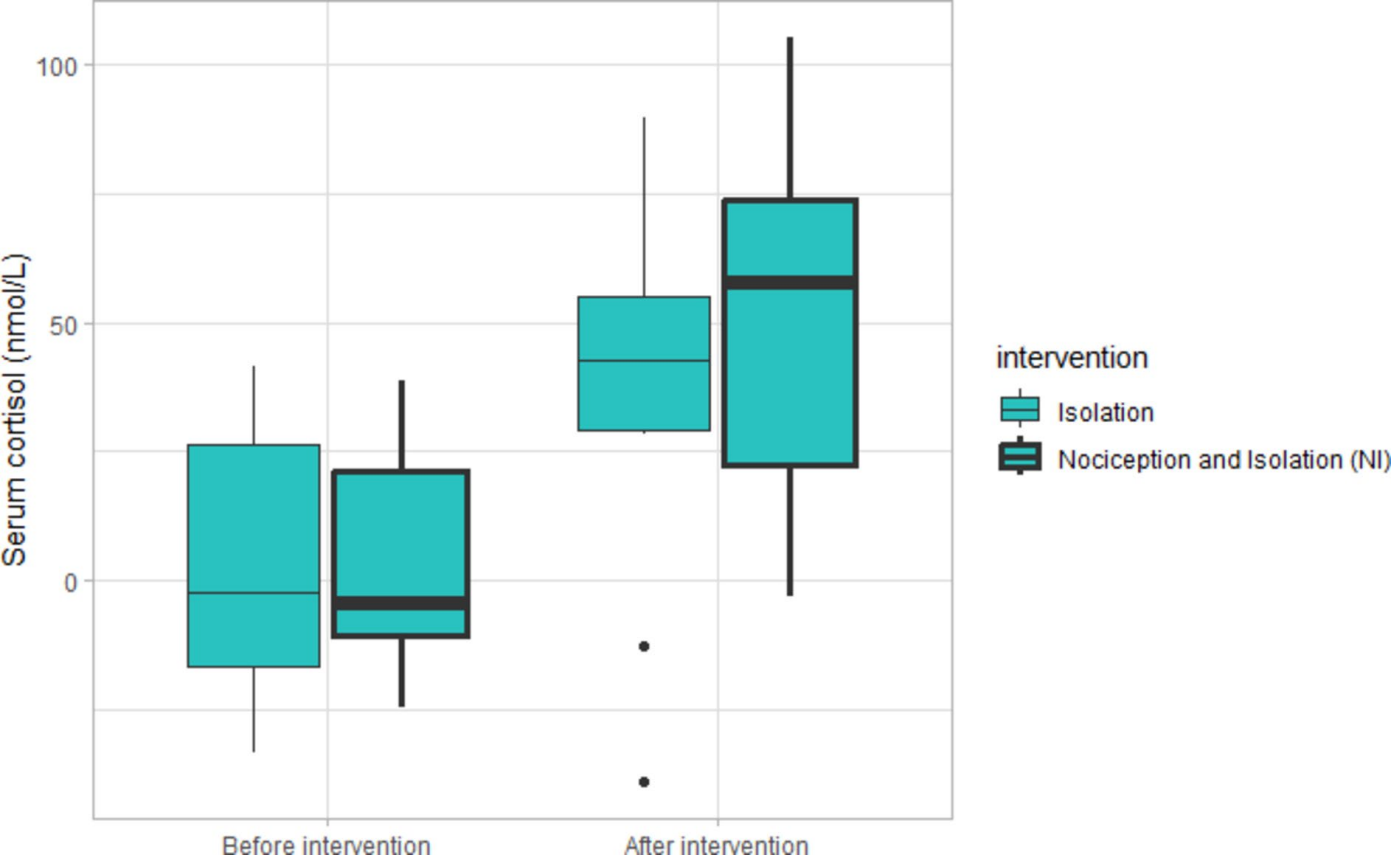
Change in serum cortisol level (*n* = 12) compared with the control in interventions involving isolation, shown as the difference from the same time of day before the intervention started (Δ). The upper and lower parts of boxes correspond to the first and third quartiles and the line corresponds to the median value. Whiskers extend to the minimum and maximum value but are no longer than 1.5 times the interquartile range. Values outside the whiskers are presented individually as larger dots.

A summary of serum cortisol levels both before and after the interventions involving isolation is shown in Fig. 4, as a confirmation of an arousal response in these interventions. The values shown are the difference (Δ) at the same time of day, to avoid an effect of diurnal variation. There was no difference between isolation alone and nociception-isolation, but cortisol level increased as a result of both interventions. Isolation increased the mean serum cortisol levels with a factor of 8.19 (t = 3.412, *p* = 0.006), while a combination of nociception and isolation increased the mean serum cortisol with a factor of 13.75 (t = 4.975, *p* < 0.001). The orders of the isolation interventions had no significant effect on the results.

### Intra- and inter-rater agreement

Intra-rater agreement for the two EquiFACS coders, based on 10% overlapping annotations in this specific dataset, was excellent, with an intra-class correlation coefficient of 0.97 (95% CI 0.97–0.98; *p* < 0.0001) for coder 1 and 0.91 (95% CI 0.89–0.94; *p* < 0.0001) for coder 2. Inter-rater agreement, calculated on 10% of the clips, was good, with an intra-class correlation coefficient of 0.78 (95% CI 0.73–0.81; *p* < 0.0001).

## Discussion

This study examined the facial activity of 12 horses during single and compound interventions involving different levels of physiological arousal, using a FACS-based coding system as a mean to identify these states. Three types of interventions were conducted: an ischemic nociceptive stimulus, isolation from conspecifics, and the administration of a non-analgesic relaxant to induce sedation. Furthermore, combinations of these interventions were examined to determine the effect of sedation or social isolation on facial activity induced by a nociceptive stimulus. Physiological markers (serum cortisol levels and heart rate) showed a high arousal response during the interventions involving isolation. Previous studies have found that heart rate has a low predictability of pain in some instances^40,41^, while other instances can produce significant changes in heart rate^42,43^. This could be due to the nociceptive stimuli inducing different levels of stress in horses and thus producing a different response in arousal. Interestingly, heart rate increased during our sedation intervention, which could be due to pulmonary vasodilation, which is a known pharmacological side-effect^35^ and not an activation of the hypothalamic–pituitary–adrenal-axis. There were no observed differences when comparing heart rate variability between the interventions except for an increase in RMSSD (Root Mean Square of Successive Differences) in the isolation intervention. This finding was unexpected because RMSSD has shown a negative correlation with horses in previous experiments^44^. As of late, RMSSD have been considered to perform more effectively during respiratory changes^45^ which was the reason for the selection of this marker since the isolation interventions were likely to induce movement and breathing changes. The isolation interventions could have produced more artefacts from movement, which has been shown to affect the signal from heart rate monitors in horses^46^ and is represented by the high range of RMSSD in the samples, particularly in the Isolation samples.

The reason for measuring physiological parameters was to validate the proposed level of arousal from the interventions. Each parameter carries its own merit when included in studies aiming to assess emotional impact, since heart rate variables and cortisol offer robust insights into the arousal part of the emotional response^47^. Solely heart rate, heart rate variability, or serum cortisol could not offer any discriminatory power when comparing the compound with the discrete intervention in this study but showed differences between low- and high arousal interventions. To understand these complex systems, both physiological and behavioral markers can contribute to greater understanding. However, the impact of movement and the quality of the collection of RR-intervals must be considered to draw correct conclusions. Cortisol data was not collected from other interventions in this study, due to the primary aim of confirming the isolation induction method but would likely have contributed to better understanding of the compound interventions and should be included in future studies. During the isolation interventions, the horses were observed at different time periods. This order was included in the statistical models for HR, RMSSD, and serum cortisol. Although the order of the isolation observations had no statistically significant effect in the models, some degree of variation was likely introduced by this study design.

Previous studies of facial expressions have examined the effect of pain^16,17,31^, isolation^11^, and sedation^18^ separately in different study populations. This study advances the field by exploring facial expressions during compound states within the same horse in a cross-over design. Using a partial least squares-based discriminant method, the frequency of facial activity could successfully discriminate between three different discrete interventions experienced by each of the 12 horses included in the study. When imposing combinations of these interventions, the model could not accurately discriminate between the interventions containing nociception from other contextual interventions. This highlights the shortcomings in using facial muscle contractions as a tool to judge whether the horse experiences a nociceptive stimulus without consideration for the possible presence of stress and sedation. The indicator variables representing isolation were inversely correlated to those representing the control and sedation and were mainly described along the axis of the t1 component in the PLS-DA plot. Thus, the main differences between these states were activity level, represented by both a higher frequency of action units and an increase in plasma cortisol levels and heart rate, where isolated horses displayed high activity levels, and sedated and neutral horses displayed low activity levels. In general, all behavioral expressions are characterized by activity level^48^, and the findings in this study indicate that this is also the case for facial activity. Since only the first component (t1) of the PLS-DA was statistically significant, it can be concluded that the discriminatory properties reflected arousal level. However, it is important to note that these interventions are not in themselves emotional states although the horses likely experience some form of emotional response during these interventions. Instead, this study demonstrates that managerial situations affect the facial activity of horses. It is currently unclear what may trigger this activity, but it could possibly include emotion^49^ and attention^44,50^.

Horses experiencing the nociception intervention in this experiment displayed certain facial expressions similar to those observed in both isolation interventions and control, suggesting that both the presence of inter-individual variation and similar patterns of AUs can be present in different states. The notion that facial expressions are prototypical for certain emotional states^51^ should therefore be considered with caution. For example, the indicator variable for the Nociception intervention could be discriminated from the baseline in the loadings plot (Fig. 2), but that individual’s scores are close to both the control and isolation scores (Fig. 1). The variation may be due to individual pain perception and display, which has been shown to be related to a multitude of factors in humans^52^ and also possibly in animals^53^. It has been reported in clinical practice, and in clinical and experimental research, that pain may be expressed from behaviors such as restlessness or pacing in certain individuals, while in others it may be expressed as introverted and depressed behavior^54^. Whether or not the horse exerts extroverted or introverted behavior is likely subject to individual variation^55^. This study’s results indicate that the same could be true for facial behaviors. Here, many physical attributes of the horse, such as breed and color, were controlled for, but earlier experiences, such as age and personality, were not accounted for, which may also partially explain the variation.

As found in an earlier study^11^, ear movements (mainly EAD101, EAD104), blinking frequency (AU145), and the dilation of nostrils (AD38) were all associated with high arousal interventions such as transportation and conspecific isolation, which had a higher frequency of AUs compared to the other interventions. Resultingly, these specific AUs tended to be discriminative, since they also dealt with much of the variation within the dataset. This should be considered as an important factor for further discussion, because these results suggest that that the sheer amount of AUs displayed during interventions with high arousal may lower the sensitivity to determine whether the horse is in pain or not. It could also explain why so few of the facial expressions observed during the nociception intervention showed significant changes (Table 2). From the PLS-DA loadings plot (Fig. 2), where the indicator variables for isolation and its compound state ended up near each other, the two states display very few differences, and the nociception component had minimal influence on the facial activity of an already isolated horse. Furthermore, during statistical testing, differences between control and the isolation interventions were only observed for ear movements (EAD101-EAD104) and nostril dilator (AD38), which were two of the most common AUs. Ears are highly expressive in horses^50^, therefore, it is credible to state that ear movements could become highly discriminatory in the frequency-based method. An exploration of co-occurring rapid facial expressions combined with slower and more substantial facial expressions of longer duration during other complex states could prove beneficial for better recognition. In the meantime, these results suggest that careful consideration is required to determine if stress is present when evaluating facial expressions in horses. The mere presence of an easily induced stressful state can produce sufficient facial activity to conceal other important facial expressions, as has been suspected previously^56^.

Pharmacological sedation is often induced to ensure greater compliance during clinical examinations in horses. The hypothesis was that facial expressions may be concealed by a muscle-relaxing effect of acepromazine^57^. The combined states of sedation and pain remained close to the control, acting as though combining these two states produced facial activity similar to that during the baseline. This means that recently sedated horses could hide facial expressions produced by the nociceptive stimulus. It has been previously reported that blinking frequency increases during stress^44^. In this study, blinking frequency (AU145) increased during all interventions including nociception or isolation, while during sedation it did not differ from control. The horses that experienced the nociceptive input displayed a higher blinking frequency despite being affected by acepromazine in sedative doses, which could be due to a physiological response of the dopaminergic effects of acepromazine^58^. Blinking type and frequency should therefore be further explored as a promising discriminatory factor in future assessment systems. This should particularly occur in equine clinical practice to detect pain in horses either with anesthesia hangover effects or under sedation, both of which could diminish other facial expressions or behaviors indicating pain. However, while blinking frequency had high discriminatory power between high arousal experiences and low- or neutral states, it had little discriminatory power within high arousal experiences. Thus, blinking frequency, and facial expressions generally, may be less reliable in situations where a high arousal state is present, such as during exercise or other stressful management procedures.

No single AUs that were discriminatory between Isolation and the compound state (NI) were found, while facial expressions during low degree nociception alone was detectable. Moreover, no single AU was discriminatory for nociception or sedation. The previous study investigating FACS during pain identified a number of facial expressions that were discriminative for pain^31^ (mainly AU101, AD38, and ear asymmetry). However, since these AUs increased in this study and were present in all interventions, they may not be discriminatory of pain per se but rather increase as a result from other stimuli. In this study a low degree of nociceptive input was assessed. However, the ischemic pain model that was used cannot measure the degree and timing of the nociception. The number of muscle contractions of the foreleg affect the resulting degree of the ischemic stimulus^59^, meaning that some of the horses in this study could have experienced less nociceptive input during the time of filming, which could explain some the variation within the dataset. The reason behind the low facial activity during the interventions with a nociceptive stimulus could simply be that a low degree of pain subsequently produces less internal stress and less facial activity. This is supported by the fact that facial expressions can be produced voluntarily, so a low degree of pain may not produce enough highly distinctive facial expressions to achieve great discriminatory power using a frequency-based method. Additionally, nociceptive thresholds can be affected by either distraction^60^ or the environment^61^ and could thus provide additional reasons for variation despite the environment being carefully controlled for in the study design.

The small number of horses included in this study is a limiting factor. However, discriminatory power was still found for this limited sample. Although PLS-DA is a relatively robust method, this dataset had great inter-individual variation, which will limit the scope for drawing further biological conclusions outside of this study. In this experimental setup, the study population consisting of 12 Standardbred trotters was homogenous in terms of breed, body, and weight, and was selected as the best mean to show that the discriminative power of facial expressions indeed diminishes when combined with other contexts. When using an exhaustive coding system, such as EquiFACS, the final dataset will inevitably contain many entries for which there are no observations, resulting in many zeroes. In the statistical analysis in this study, these were handled as true zeroes. A typical form of error in behavioral studies that share approximately the same Poisson distribution as the present study is a structural error, such as too short an observation window, which may be the reason for certain facial behaviors not being observed. Here, 30-s videoclips were analyzed, which is a much longer observation period compared to previous studies, some of which have analyzed still frames extracted from video sequences^16,62^. Although analyzing images can be beneficial for analyzing grimaces, EquiFACS relies on temporal definitions (time of onset – time of offset). Based on earlier EquiFACS studies^11,31^, 30 s is deemed to be a sufficient sample to draw valid conclusions from, but it is important to acknowledge that longer observation windows could produce different results. On the other hand, longer observation periods pose challenges in controlling the external environment, animals habituating to the situation, and controlling for the actual response to the intervention.

The results in this study pave the way for refining and comparing methods for future evaluation of pain, stress, and sedation. As shown, simple methods can be sufficient to discriminate between experiences thought to affect valence and arousal in horses. It has been recommended that studies such as this should always include a control group that is free of pain^63^. The results here indicate that this needs to be taken one step further and environment and other factors, such as impact due to contextual situations, should be controlled for when evaluating facial expressions.

## Conclusions

This experimental study successfully discriminated between non-compound interventions of conspecific isolation, sedation, and low-degree nociceptive input using PLS-DA based on EquiFACS data. When combining the interventions, facial expressions during conspecific isolation could not be differentiated from facial expressions produced by a nociceptive stimulus. Likewise, facial expressions produced by a nociceptive input in combination with a sedative drug (acepromazine) could not be differentiated from the control. However, differences in the frequency of the facial action unit blinking (AU145) in the state of “sedation” and “sedation combined with nociception”, indicated that blinking frequency could be a future method for further investigation. Careful consideration must be given when evaluating facial expressions in horses during challenging situations, notably in complex situations where the context is unknown.

Methods.

### Animals

Ethical permission for the use of horses in this study was obtained from the Ethics Committee for Animal Experiments in Uppsala, Sweden (Approval no. 5.8.18–10767/2019) in accordance with Swedish law and the experiments were performed in accordance with ARRIVE guidelines. All horses were owned by the research facility and both permission to use the horses and the samples obtained from these horses were acquired prior to experimentation. Twelve horses (three geldings and nine mares) participated in the study with a mean age of 14 years (SD = 4.0) and a range of 7–20 years. The horses were all Standardbred trotters, either brown or dark brown in color, with a mean body weight of 554 kg (SD = 51.9) with a range of 486–652 kg. The horses were kept at the teaching facility for at least six months prior to the study and were part of the teaching herd at the university. Within the facility, they were usually stabled overnight in boxes measuring 3 m x 4 m and were kept in paddocks or pasture enclosures for at least 6 h in small herds between approximately 07.30 and 15.00. They were fed hay four times a day and oats once daily with automatic dispensers and there was therefore no involvement of facility staff.

### Study preparations

Horses were divided into pairs which were normally stabled in neighboring boxes or shared an outside enclosure herd. Additionally, they were deemed as social companions by their caretakers. These two horses were observed simultaneously over 6 experimental periods over 3 experimental days each, but each had their own randomized order in the cross-over. For at least three days before experimentation, two horses at each experimental period were habituated to other boxes in the same facility. These boxes were placed in a closed off part of the facility to eliminate disturbances from factors outside of the experiment, such as facility staff, and only the same three operators were in the closed off part during experimentation and facility staff was instructed to keep the area undisturbed from other horses and people. The two horses had full visibility of each other but not of the surrounding area outside the sectioned stable. Feeding or housing routines were unchanged during the habituation period and the horses were handled by the regular staff during the habituation period. The included horses underwent clinical examinations by a veterinarian and were considered healthy before experimentation. The horses also experienced no changes to their feeding routines during the experiments. Housing routines remained largely the same but there was a 2-hour reduction in pasture time due to the experimentation. Furthermore, to prevent contamination to the intravenous catheter, horses were kept in individual paddocks with visual contact with conspecifics throughout the three experimental days.

At least one hour before the start of the experiment, an area on the neck of each horse was clipped and sterilized, and an intravenous catheter (Mila cannula, 2,7 × 130 mm; MILA International Inc., Florence, USA) was placed in the jugular vein to administer sedation and draw blood samples, thereby reducing the number of venous punctions. The catheter was sutured in place intradermally to secure it during the experiments and was removed directly after the last intervention on the last day. If accidentally displaced during the experiments, a new catheter was fitted using the same procedure. Prior to the observations, all horses, regardless of intervention, were fitted with a pneumatic blood pressure cuff, which was placed directly above the carpus on a random foreleg. During the catheter placement the horses were restrained using a rope and halter.

### Study design

**Fig. 5:**
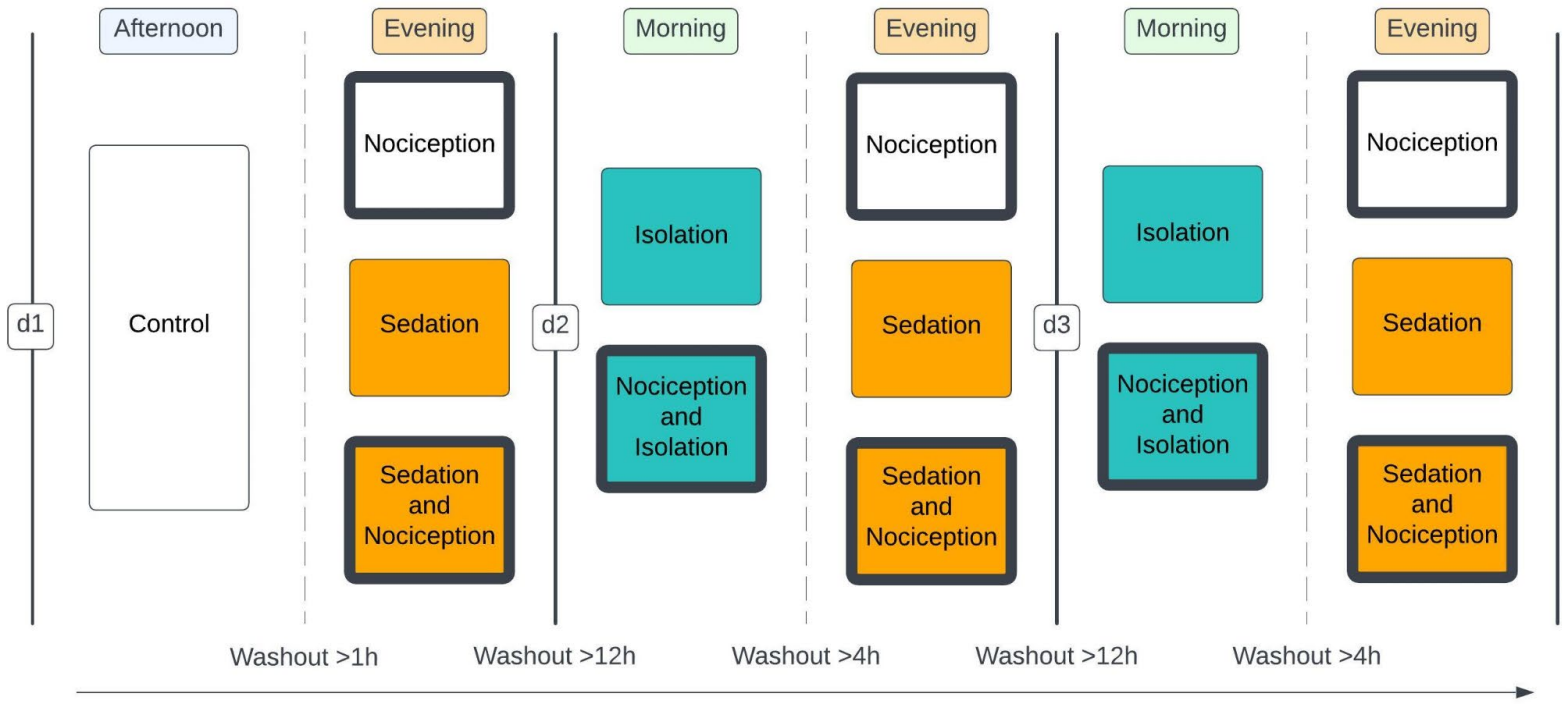
Schematic diagram of the semi-randomized cross-over design. Two interventions were performed each day (d1–d3) for each horse (*n* = 12) with a minimum washout before each intervention, as stated in the figure.

Behaviors and facial expressions of all 12 horses were observed during five experimental interventions: nociception (N), isolation (I), pharmacologically induced sedation (S), combined nociception and isolation (NI), and combined nociception and pharmacological sedation (SN). The interventions were arranged in a semi-randomized cross-over design (Fig. 5). The physiological response to an induced psychological state of sensory alertness (arousal), was measured using physiological markers, to measure the proposed effect of the interventions.

The horses were video filmed throughout the entirety of the interventions (15–20 min) while unrestrained in their box, and without an observer present. The interventions involving nociception and sedation occurred during the evening and the interventions involving isolation occurred during the morning. Sections outside the boxes were quiet and no people or horses passed by during the experiment. Horses were only handled by the same three operators throughout the whole experiment (J.L, S.H.R., and H.B.), who took extra consideration into handling the horses as little as possible. The order of inductions was randomized, and this was done so that each possible order (*n* = 6) of the interventions occurred for two horses each, minimizing the effect of the order of the interventions. The interventions took place at the same time each day, to minimize the influence of facility routines and diurnal variations in serum cortisol levels. Evening interventions (N, S, SN) were carried out simultaneously on the two horses while morning interventions (I, NI) were carried out within one hour from each other in a randomized order with a minimum of 30 min between the interventions.

### Control (C)

In order to gain a control to compare against, a recording of the horse’s facial expressions was performed during a sham intervention where the horse was left undisturbed in their box thereby mimicking all experimental protocol without further intervening with the horse. The control was performed before all other interventions to reduce expectancy behaviors from the horse and to maximize the wash-out between the other interventions. The horse was fitted with the blood pressure cuff and had their catheter placed before the recording of facial expressions took place. The horse’s companion was left undisturbed for all interventions except for isolation.

### Nociception intervention (N)

The horse was haltered and a mild temporary ischemic nociceptive stimuli, previously determined as a model of pain^64^, was induced using the pneumatic blood pressure cuff fitted on the foreleg of the horse. The cuff was inflated to 240 mmHg and fastened using an elastic wrap before inflating, causing the cuff to expand inward. The induction lasted for a maximum of 20 min while the horse was left alone in the box, before the horse was haltered again and pressure was relieved.

### Sedation intervention (S)

Horses were haltered and pharmacological sedation was induced using acepromazine, a derivate of phenothiazin (Plegicil vet., 10 mg/ml, Pharmaxim, Helsingborg, Sweden), via intravenous injection through the permanent catheter in the jugular vein. Acepromazine causes pharmacological sedation but does not have an analgesic effect in horses^65,66^. A specific dosage was calculated for each individual horse according to the manufacturer’s recommendations for moderate to heavy sedation (0.075 mg/kg). The horses were left alone in the box and given an onset with a minimum of 20 min after injection before data collection commenced.

### Isolation intervention (I)

The isolation intervention consisted of a change in the horse’s daily routine, in which a horse was kept in the stable, expecting to be released in their outside enclosure. The first conspecific isolation was performed between 08.00 and 09.00 in the morning, when the horses were habituated to going outside for the day. The horse was left alone in the box while their companion horse was brought directly outside the sectioned stable but still in a closed off part of the facility, leaving the stabled horse alone without visual input from conspecifics. After the companion horse was brought out, the stable was closed off and the stabled horse was left alone without observers unrestrained in the box for 20 min until the companion horse was returned. After a minimum washout of 30 min the intervention was repeated on the other horse between 09.00 and 10.00. The order of horses was randomized.

### Compound interventions (NI and SN)

Compound states were induced by a combination of the above interventions. In the intervention SN, pharmacological sedation was induced according to a protocol that involved sedation first, a 20-minute period of onset for the drug, and then nociception. In the intervention NI, the pressure in the pneumatic blood pressure cuff was applied first and the isolation was performed immediately afterwards.

### Video collection and processing

All facial expressions during the interventions were recorded on video using four wall-mounted surveillance cameras with night vision (WDR EXIR Turret Network Camera, HIKVISION, Hangzhou, China). Ambient lighting was provided by nine standard fluorescent lights mounted on the ceiling on a standardized and regulated schedule. Lighting from the outside was limited, as the walls hindered external light into the stables. The cameras were mounted in each corner of the box, which ensured that the video would capture the horse from four angles simultaneously.

A video was recorded for the full 15–20 min intervention and recordings were exported at 1080p resolution and run through automatic face detection software^67^, which calculated the probability of the horse’s face being visible every 5 s throughout the whole video. Video segments of 30 s from each intervention and horse were selected for annotation based on the results of the face detection software. Segments within the range of 9–11 min after the intervention had started with the highest probability of a horse face being visible were selected for annotation.

The 30 s segments were annotated using the Equine Facial Action Coding System (EquiFACS) by two certified coders, where 10% of the clips were scored by both coders and used to calculate inter-rater agreement and 5% of the clips were scored by the same coder twice to calculate intra-rater agreement and the coders were blinded depending on which intervention the horse underwent. The observations were generated using the open software ELAN (Nijmegen: Max Planck Institute for Psycholinguistics, The Language Archive, version 5.4)^68^, where onset, offset, and duration of each code in EquiFACS were annotated on the video timeline, which provided a frequency and duration for each of the facial expressions.

### Heart rate parameters

Inter-beat intervals during all interventions were sampled using a remote-controlled transmitter (Polar Wearlink (Polar Electro OY, Kempele, Finland) together with a controlling unit (Polar RS5800CX). This allowed for measurements of R-R intervals constantly throughout the interventions, without the interference of an observer. Since heart rate is commonly used when evaluating clinical aspects of pain, this variable was deemed interesting to analyze. Root Mean Square of Successive Differences (RMSSD) was included as a complement because it is considered to be a stable marker for parasympathetic nerve activity (PNS) and particularly suited for short-term samples^69^. HRM files containing R-R intervals were analyzed using Kubios HRV Premium (version 3.5.1, Kubios Oy, Kuopio, Finland) using the in-built automatic artifact beat correction and exported as mean and range for each horse and intervention.

### Blood sampling

Blood samples were taken before and after the interventions involving isolation at 08.00 and 09.00 or at 09.00 and 10.00, depending on which time the intervention took place. Since cortisol has a diurnal baseline^70^, samples were taken at the same times on day 1, in order to have a baseline for comparison. The blood samples on day 1 were taken through a puncture of the jugular vein using a vacutainer needle, to minimize the time in which the horse had an intravenous catheter, while samples during the experimentation involving interventions were drawn from the permanent catheter. When drawing blood from the catheter, the first 100 mL of blood were discarded, to avoid contamination from the catheter, and samples were drawn into standard serum tubes. Samples were compared to their respective time when the Isolation and NI-intervention took place, respectively.

The samples were left in a refrigerator for at least 30 min to allow the clot to separate, after which they were centrifuged at 5000 RPM for 10 min. Plasma and serum were then aliquoted and frozen at -80 °C until analysis. Serum cortisol was analyzed in two replicates, using a commercial immunoassay instrument (Immulite 2000XPi, Siemens Healthcare Diagnostics) with a reagent Veterinary Cortisol, lot 127.

### Statistical analysis

A partial least squares-discriminant analysis was performed in SIMCA^®^ (version 17) for Windows (Sartorius Stedim Data Analytics AB, Umeå, Sweden), using standard settings. This analysis allowed for the selection of discriminatory Action Units (AUs). Interventions were set as class variable, all AUs were set as variables, and individual horses were set as secondary class variable. The first two components were included in the analysis. Plotting of the PLS-DA analysis was completed using “ggplot2”^71^ in R.

Statistical analysis was conducted in R^72^. Zero-inflated generalized linear mixed models within a Poisson distribution using the package “glmmTMB”^73^ were built to make inferences about individual AUs and were compared to other distributions. This model generated the best fit with the lowest Akaike information criterion. Intervention, sex, and age were set as a fixed factors and horse was set as a random factor. χ^2^-test output for different EquiFACS codes was obtained in a type III analysis of variance (ANOVA) and calculated on 5 degrees of freedom. Estimated marginal means were investigated using the “emmeans”^74^ package and contrasts were compared between baseline and intervention. *p*-values were corrected for multiple comparisons using the multivariate t distribution. Statistical testing for the heart rate, RMSSD, and serum cortisol was performed by fitting linear mixed models using the package “lme4”^75^, with horse as a random variable and intervention as a fixed variable. For the isolation interventions, the random order within the pair of horses was included as a fixed factor, as the order of the pairs could influence the results. Contrasts were tested using “emmeans”^74^. Both inter- and intra-rater agreement was estimated by intra-class correlation coefficients calculated using the package “irr”[76] based on the 10% of clips annotated by both coders (inter) and the same coder twice (intra). A two-way mixed effects model based on a single rater and strict agreement was used.

## Electronic supplementary material

Below is the link to the electronic supplementary material.

Supplementary Material 1

Supplementary Material 2

## Data Availability

All data generated and analyzed during this study is included in this published article and its supplementary information files.
