# Supplementary material for: Facial expressions during compound interventions of nociception, conspecific isolation, and sedation in horses
Source: Sci Rep. 2025 Feb 13;15:5373. doi: 10.1038/s41598-025-89329-x (PMC11825850; doi:10.1038/s41598-025-89329-x)
Supplement: Supplementary file 1 — Supplementary Material 1 [file 41598_2025_89329_MOESM1_ESM.pdf]

Supplementary file

Facial expressions during compound interventions of nociception, conspecific isolation, and sedation in horses

Johan Lundblad*, Marie Rhodin, Elin Hernlund, Hanna Bjarnestig, Sara Hidén Rudander, Pia Haubro Andersen

Dept. of Animal Biosciences. Swedish University of Agricultural Sciences. Uppsala, Sweden.

List of Action Units and their meaning. Based on the manual by Wathan et. al. (2014).

Wathan, J., Burrows, A. M., Waller, B. M. & McComb, K. EquiFACS: The equine facial action coding system. PLoS ONE 10, e0131738; 10.1371/journal.pone.0131738 (2015).

**Action Unit**	**Meaning**
EAD101	Ears forward
EAD102	Ear adductor
EAD103	Ear flattener
EAD104	Ear rotator
AU101	Inner brow raiser
AU143	Eye closure
AU145	Blink
AU47	Half blink
AU5	Upper lid raiser
AD1	Eye white increase
AU10	Upper lip raiser
AU12	Lip corner puller
AU113	Sharp lip puller
AUH13	Nostril lift
AU16	Lower lip depressor

AU17	Chin raiser
AU18	Lip pucker
AU122	Upper lip curl
AU24	Lip presser
AU25	Lips part
AU26	Jaw drop
AU27	Mouth stretch
AD160	Lower lip relax

AD19	Tongue show
AD29	Jaw thrust
AD30	Jaws sideways
AD133	Blow
AD38	Nostril dilator
